# Identifying Markers of Cardiovascular Event-Free Survival in Familial Hypercholesterolemia

**DOI:** 10.3390/jcm10010064

**Published:** 2020-12-27

**Authors:** Etienne Khoury, Diane Brisson, Nathalie Roy, Gérald Tremblay, Daniel Gaudet

**Affiliations:** 1 Lipidology Unit, Community Genomic Medicine Center, Department of Medicine, Université de Montréal, ECOGENE-21 Clinical and Translational Research Center, Chicoutimi, QC G7H 7K9, Canada; etienne.khoury@umontreal.ca (E.K.); diane.brisson@ecogene21.org (D.B.); nroy1111@outlook.com (N.R.); gc.tremblay@videotron.ca (G.T.); 2 Lipid Clinic, Chicoutimi Hospital, Chicoutimi, QC G7H 5H6, Canada

**Keywords:** familial hypercholesterolemia, cardioprotective markers, septuagenarians, cardiovascular events

## Abstract

Familial hypercholesterolemia (FH) is an autosomal dominant trait characterized by elevated low-density lipoprotein-cholesterol (LDL-C) concentrations appearing at birth and is associated with increased risk of premature atherosclerotic cardiovascular disease (CVD). However, in some cases, FH subjects over 70 years of age have surprisingly never experienced any CVD symptoms throughout their entire lives. The objective of this study consists of identifying biological and environmental markers acting as cardioprotective factors and associated with unexpected survival in FH. Upon age and reported cardiovascular events (CVE) stratification, we identified a total of 458 French–Canadian FH subjects with premature reported CVE, and 1297 young adults as well as 24 elderly subjects (≥70 years) who have never reported CVE requiring hospitalization. Logistic regression models were used to depict cardioprotective markers among FH survivors (≥70 years). Regression analyses of the FH cohort showed that female sex (odds ratio (OR) = 12.92 (4.23–39.46); *p* < 0.0001), high levels of high-density lipoprotein (HDL)-C (OR = 6.76 (2.43–18.79); *p* = 0.0002) and elevated concentrations of adiponectin (OR = 71.40 (5.20–980.47); *p* = 0.001) were significant contributory factors in reducing FH-related CVD risk. Notably, female (OR = 11.45 (1.25–105.98); *p* = 0.031) and high HDL-C (OR = 9.78 (1.75–54.67); *p* = 0.009) were shown to be significant covariates associated with survival in FH. Non-smoking (OR = 11.73 (4.36–31.56); *p* < 0.0001) was also identified as an environmental factor associated with CVE-free survival. Based on this configured model of premature CVE occurrence, these results demonstrated that, beyond LDL-C levels, female sex, high HDL-C, elevated adiponectin and non-smoking are important markers that contribute to a reduced risk of CVD and CVE-free survival in FH.

## 1. Introduction

Familial hypercholesterolemia (FH) is an inherited autosomal dominant trait characterized by an elevated plasma low-density lipoprotein-cholesterol (LDL-C) concentrations and increased risk of severe and premature coronary artery disease (CAD) [1,2]. Epidemiological studies have recently reported that the prevalence of the heterozygous form of FH (HeFH) is estimated around 1:300 [3,4] and the homozygous form (HoFH) around 1:160,000 to 300,000 individuals [5]. However, this prevalence tends to be higher in some founder populations, such as Afrikaners, Christian Lebanese and French–Canadians [6]. This congenital metabolic disease largely occurs due to defects in genes coding for LDL receptor (*LDLR*), and less frequently, apolipoprotein B (*APOB*), proprotein convertase subtilisin/kexin type 9 (*PCSK9*) and LDL receptor adaptor protein (*LDLRAP1*) [7,8]. Regardless of the FH phenotype severity that generally varies between FH-causing mutations, high-intensity hydroxymethylglutaryl co-enzyme A (HMG CoA) reductase inhibitors (statins) therapy is highly recommended in asymptomatic atherosclerotic cardiovascular disease (ASCVD) if LDL-C > 4.9 mmol/L (190 mg/dL) [9] or whenever LDL-C levels remain above 1.4 mmol/L (>55 mg/dL) and less than 50% reduction from baseline are yet achieved in FH patients with ASCVD, who are considered at very high risk. Combination therapy is also recommended if the treatment goal is not achieved [10].

If left untreated, young adult patients (20–39 years old) with FH will suffer from 100-fold increased risk of mortality caused by coronary heart disease, and those with HeFH will experience cardiovascular events (CVE) by the age of 60 [11,12,13,14]. In fact, without treatment, approximately half of FH individuals will survive to 60 years and only 20% to 70 years [15]. Studies have also demonstrated that inadequate treatment of FH can lead to increased risk of coronary events by age 50 (50% in men and 20% in women) [11,16]. However, characteristics and prevalence in CVE can remarkably vary between FH, even among those who share the same mutation [17]. Therefore, we believe that FH clinical manifestations and its cardiovascular outcomes are most probably driven by multiple players, including genetic, environmental and metabolic factors [18].

Based on our French–Canadian cohort, several FH individuals have survived without reporting any CVE past 70 (even 80) years of age. This is extremely surprising knowing that many of these patients are sub-optimally treated and that effective FH treatments, including statins, have been available for less than 35 years. We thus hypothesize that some patients with FH may present cardioprotective characteristics that promote survival. The main objective of this study is to identify markers of CVE-free survival or atherosclerotic-resistant factors in FH that could potentially lead to important targets for cardiovascular disease (CVD) prevention in the general population.

## 2. Materials and Methods

### 2.1. Subjects

**Figure 1:**
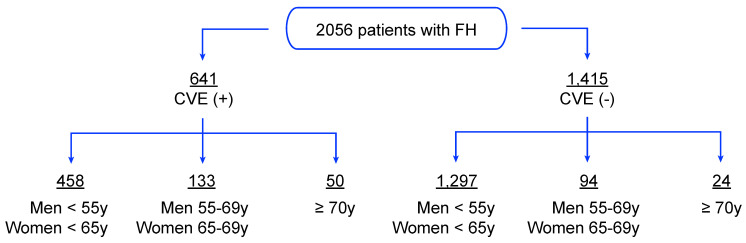
Study design. Representation of the initial sample (*n* = 2056) of French–Canadian subjects with FH, among them 1116 subjects whose FH status was confirmed by Simon Broome criteria and 940 definite FH adults known to carry FH-causing mutations in the *LDLR* gene. The study sample was stratified according to age and occurrence of cardiovascular events (CVE) requiring medical intervention (myocardial infarction, unstable angina, coronary artery bypass grafting, percutaneous transluminal coronary angioplasty).

The initial sample consists of 2056 French–Canadian FH patients, among them 1116 subjects whose FH status was confirmed by Simon Broome criteria and 940 definite FH adults known to carry FH-causing mutations in the *LDLR* gene, mainly the identical by descent (IBD) heterozygous carrier for the defective French–Canadian type 2 (W66G) mutation. The Simon Broome diagnostic criteria for FH were used to either identify definite FH (i.e., plasma LDL-C > 190 mg/dL (4.9 mmol/L) plus, either DNA-based evidence of an LDLR mutation or the presence of typical tendinous xanthomata, or tendon xanthomas in first- or second-degree relative) or possible FH (i.e., plasma LDL-C > 190 mg/dL (4.9 mmol/L) plus, either family history of myocardial infarction (MI) in the first-degree relative (<60 years) or second-degree relative (<50 years) or family history of elevated total cholesterol greater than 290 mg/dL (7.5 mmol/L) in the first- or second-degree relatives). During their first visit at the Chicoutimi lipid clinics, all FH patients were evaluated by a multidisciplinary team and data regarding lipid profile, plasma adiponectin concentrations, treatment regimen and other factors of metabolic syndrome (Type 2 diabetes (T2D), hypertension) were collected from those who agreed to participle in this study, as described elsewhere [19]. Only Lp(a) levels were collected when data were first available in the patient’s medical file. Subjects were stratified according to age and occurrence of CVE requiring hospitalization (myocardial infarction, unstable angina, coronary artery bypass grafting, percutaneous transluminal coronary angioplasty), as follows: group 1 for CVE(+) in men <55 years and women <65 years; group 2 for CVE(-) in men <55 years and women <65 years; group 3 for CVE(-) in men and women ≥70 years (Figure 1). The presence of CVE was collected as documented in the patient’s medical file, whether the event occurred prospectively or retrospectively of the analyzed biological and environmental data. In fact, around 35% of patients who experienced CVE had never been evaluated at the lipid clinic before their cardiovascular event; however, all subjects have been followed at the clinic since enrollment (i.e., mid-1990s), and data presented in this study regarding incident events were collected until 2011. Those who did versus did not report CVE were classified, respectively, as CVE (+) versus CVE (-). Across each of these selected groups (1, 2 and 3), the number of patients (*n* = 208, 681 and 6) with confirmed FH-causing mutations in the LDLR (C646Y, D>15KB, E207K, R329X, W66G and Y468X) was highlighted, respectively. Lipid-lowering treatment in FH cohorts was classified as a percentage of subjects who were treated with a lipid-lowering therapy versus naïve patients. T2D was diagnosed in patients whose either fasting glucose > 5.6 mmol/L or reporting drug treatment for elevated glucose and hypertension was classified as blood pressure ≥  130 mmHg or  ≥  85 mmHg for systolic or diastolic blood pressure, respectively [20]. Notably, all patients who did not meet the FH status using the criteria mentioned above were excluded from the study and age groups (CVE(+): 133 men 55–69 years and women 65–69 years and 50 ≥ 70 years; CVE(-): 94 men 55–69 years and women 65–69 years) were also excluded from the statistical analyses as data according to each of these groups would not provide additional information to the established hypothesis and did not fit the study design. All subjects were screened at the Chicoutimi Hospital Lipid Clinic or ECOGENE-21 Clinical Research Center and agreed to participate in studies on genetic determinants of T2D, CAD and dyslipidemia. Subjects gave their informed consent to participate in this study and were assigned a code that systematically de-identifies all clinical data [21]. They were selected to be included in the present study based on the availability of data on lipid-associated parameters, adiponectin levels, smoking habits and daily life stress assessment.

### 2.2. Blood Samples

Blood samples were obtained after a 12-h overnight fast. Cholesterol and triglycerides (TG) were measured by enzymatic assays. LDL-cholesterol levels were calculated using the Friedewald formula, unless TG levels were >4.5 mmol/L. Ultracentrifugation was performed to measure the cholesterol content of very low-density lipoprotein (VLDL), LDL and high-density lipoprotein (HDL) particles. Apolipoprotein (Apo) B and Apo A1 were measured using nephelometry. Adiponectin concentrations were determined by ELISA assays (B-Bridge International, Inc., San Jose, CA, USA) as well as Lp(a) levels.

### 2.3. Lifestyle Factors

Smoking habits were classified as non-smokers (never smoked or sporadic smoking: <5 cigarettes/day ceased >10 y ago) versus smokers. Daily life stress was documented using a questionnaire and classified as low-to-moderate versus high-to-extreme.

### 2.4. Statistical Analysis

**Figure 2:**
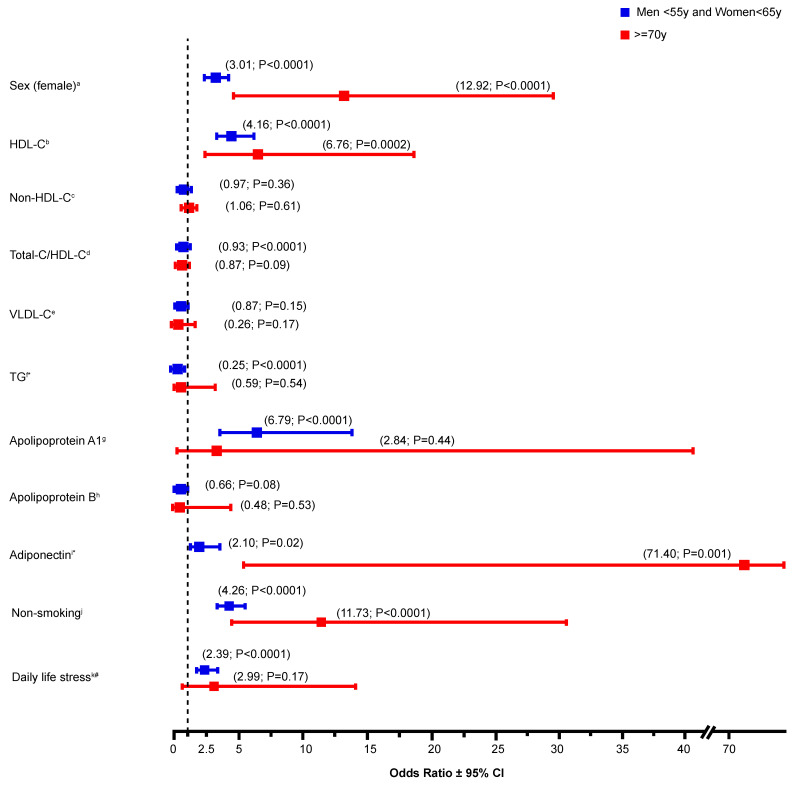
Univariate analysis of the contribution of biological and environmental variables to the relative odds of CVE-free survival in FH patients. Data represent the odds ratio of sex (female), various lipid parameters as well as non-smoking and daily life stress (low-to-moderate) on cardiovascular events (CVE)-free survival. Lipid-lowering drug treatment was included in all models. Statistical significance is defined with a *p*-value < 0.05. * Plasma adiponectin and triglycerides values were log_10_-transformed. ^#^ As compared to high-to-extreme stress. ^a^ (*n* = 458 (group 1), *n* = 1297 (group 2); *n* = 24 (group 3)); ^b^ (*n* = 457 (group 1), *n* = 1292 (group 2); *n* = 24 (group 3)); ^c^ (*n* = 457 (group 1), *n* = 1289 (group 2); *n* = 24 (group 3)); ^d^ (*n* = 457 (group 1), *n* = 1289 (group 2); *n* = 24 (group 3)); ^e^ (*n* = 116 (group 1), *n* = 362 (group 2); *n* = 17 (group 3)); ^f^ (*n* = 457 (group 1), *n* = 1294 (group 2); *n* = 24 (group 3)); ^g^ (*n* = 223 (group 1), *n* = 472 (group 2); *n* = 8 (group 3)); ^h^ (*n* = 219 (group 1), *n* = 628 (group 2); *n* = 9 (group 3)); ^i^ (*n* = 268 (group 1), *n* = 695 (group 2); *n* = 12 (group 3)); ^j^ (*n* = 453 (group 1), *n* = 1172 (group 2); *n* = 22 (group 3)); ^k^ (*n* = 289 (group 1), *n* = 1009 (group 2); *n* = 15 (group 3)).

Based on the initial sample, statistical analyses of the lipid profile was performed by comparing CVE-free survivors (CVE(-) from groups 2 and 3) to those at younger age with confirmed premature CVE (CVE(+) from group 1) (Table 1). Categorical variables were compared using the Pearson χ2 statistics or the Fisher’s Exact test statistics, whereas group differences for continuous variables were compared with one-way Anova followed by Bonferroni post-hoc tests. Variables with skewed distributions were log_10_-transformed before statistical analysis. Univariate logistic analyses were performed on variables that showed significant differences in prevalence (e.g., sex), mean +SD (e.g., HDL-C) or median (interquartile range (IQR)) (e.g., triglycerides) between CVE(+) and CVE(-) groups (Figure 2). Among these identified biological and environmental markers that revealed significant association with reduced risk of CVD in FH subjects, multivariate logistic regression models were built in order to calculate the relative odds (odds ratio (OR)) of CVE-free survival and investigate their contribution as cofactors within each corresponding model (Table 2 and Table 3). For example, “Model 1” was simply defined as one of the univariate models (i.e., sex), and then additional covariates were added, such as HDL-C for “Model 2” and Apo A1 for “Model 3”, as indicated in Table 2. Current lipid-lowering treatment was also added as a covariate to the regression analyses in all models and categorized into four quartiles of possible dose for each statin, ranging from quartile 1 (low dose) (e.g., rosuvastatin 5 mg, atorvastatin 10 mg) to quartile 4 (high dose) (e.g., rosuvastatin 40 mg, atorvastatin 80 mg), taking into consideration combination therapy (e.g., quartile 4 for statin + ezetimibe). Patients with missing data have been excluded from statistical analyses and the number of patients considered for each variable comparison was mentioned in the table legend. *p*-values were two-sided. Statistical significance was considered when *p* < 0.05. All statistical analyses were performed with the SPSS package (release 21.0, SPSS, Chicago III).

## 3. Results

### 3.1. Biological Factors Associated with CVE-Free Survival in FH

In order to investigate the contribution of biological markers to the cardiovascular events occurrence in patients with FH, group comparison was performed between adult patients who reported premature CVE at young age (men <55 years and women <65 years) (group 1) (*n* = 458) versus CVE(-) elderly patients (≥70 years) (*n* = 24) (group 3), who have surprisingly never experienced any CV symptoms, and are thus considered to be true survivors. Selecting extreme phenotypes, despite the relatively small sample size, would represent an interesting strategy to depict cardioprotective markers in the FH cohort. In addition, we thought that adding to the statistical analyses young adults without reported CVE (*n* = 1297) (group 2) would also be interesting for two main reasons: a first one is to highlight markers under an association analyses performed within the same group of age, where group comparisons (i.e., group 1 versus 2) could reveal parameters also considered as cardioprotective markers. The second is to support our findings by showing how these identified markers tend to have a greater impact across CVE(-) groups, especially when it comes to the cohort of unexpected survival.

Sex distribution between groups was significantly different, as women occupied around 60% of group 2 (CVE(-) in men <55 years and women <65 years) and more than two third (83%) of patients in group 3 (CVE(-) FH survivors (*p* < 0.0001)) (Table 1), whereas the prevalence of CVE in the comparison group (group 1: premature CVE(+)) was more than two times higher in men (70%) than women (30%). In fact, female sex contributed to the survival of CVE(-) FH subjects in elderly groups (OR = 12.92 (4.23–39.46); *p* < 0.0001) (Figure 2). With regard to the lipid parameters, data showed that HDL-C and Apo A1 levels were significantly higher in group 2 when compared to the premature CVE-reported group, whereas, Non-HDL-C, Total-cholesterol (TC)/HDL-C ratio, VLDL-C, and TG levels were lower. Similar trends were also observed in the elderly group 3, where HDL-C was by far the most notable marker associated with CVE-free survival, showing a 23% increase in its plasma concentration when compared to the premature CVE(+) group. Interestingly, adiponectin, which is known to act as a cardioprotective hormone due to its anti-inflammatory and anti-oxidant characteristics [19,22,23], also showed higher levels in CVE-free survivors. Not to mention that decreased levels of Lp(a) were not revealed as markers of unexpected survival in elderly patients, due to missing data. However, despite not reaching the statistical threshold, Lp(a) levels in the young adult CVE(-) group 2 tend to be decreased when compared to group 1 (24.40 versus 19.20 mg/dL) (Table 1), an association known to be highly present between this lipoprotein and CVD risk assessment in FH.

In order to evaluate the contribution of these identified biological variables to the odds of CVE-free survival in the FH cohort, regression analyses were performed. Data presented in Figure 2 showed that both high HDL-C (OR = 6.76 (2.43–18.79); *p* = 0.0002) and elevated adiponectin (OR = 71.40 (5.20–980.47); *p* = 0.001) levels contributed to the CVE-free survival in elderly patients (≥70 years). Moreover, high concentrations of HDL-C (OR = 4.16 (2.82–6.15); *p* < 0.0001), Apo A1 (OR = 6.79 (3.31–13.96); *p* < 0.0001) and adiponectin (OR = 2.10 (1.13–3.88); *p* = 0.02) were depicted as important contributory factors associated with a reduced risk of CVD in young FH subjects (men <55 years and women <65 years).

Multivariate analyses also revealed that female sex (group 3; Model 2: OR = 11.45 (1.25–105.98); *p* = 0.031) and high HDL-C (group 3; Model 2: OR = 9.78 (1.75–54.67); *p* = 0.009) were revealed as cofactors that are positively associated with CVE-free survival in FH (Table 2). The type and dose (in quartiles) of lipid-lowering drugs were included for all CVE survival-related models. Notably, 73.6% of patients with reported premature CVE were following a lipid-lowering regimen (mostly statins and ezetimibe), whereas 55.5% of the young adult CVE(-) group and 58.3% of elderly patients with CVE (-) were treated with a lipid-lowering therapy. Most FH patients were classified as quartile 1 category of dose for each statin across all three cohorts. As expected, men <55 years and women <65 years with CVE(+) accounted for the majority of patients with additional cardiovascular risk factors, such as T2D (9.8%) and hypertension (35.8%) when compared to CVE(-) groups except that hypertension seems to be more abundant in elderly patients, a phenomenon known to be mostly associated with aging (Table 1).

### 3.2. Environmental Factors Associated with CVE-Free Survival in FH

Other than identifying biological markers involved in the CVE-free survival in FH, we also thought to study the contribution of environmental factors to the relative odds of CVE-free survival in FH patients. Non-smoking (OR = 11.73 (4.36–31.56); *p* < 0.0001) showed a contributory effect on CVE-free survival in elderly patients (group 3) (Figure 2). The impact was even more pronounced in the FH survivors than young adults from the CVE (-) group. In fact, non-smoking (OR = 4.26 (3.28–5.51); *p* < 0.0001) as well as daily life stress (low-to-moderate) (OR = 2.39 (1.73–3.31); *p* < 0.0001) were shown to play an important role in the CVD risk management in FH (group 2) (Figure 2). Both environmental markers were also revealed as cofactors positively associated with reduced risk of CVE in young FH (men <55 years and women <65 years) (Table 3).

## 4. Discussion

### 4.1. Biological Markers of Survival

This study, which was designed to compare CVE(-) elderly subjects (≥70 years) to premature CVE(+) in young adults, reveals biological and environmental markers of survival in the FH population. It is well established that CVD risk in FH is modulated by several genetic and environmental factors [24]. Here, we showed that gender and several lipid parameters could be classified as factors that predict survival in FH patients. More precisely, HDL-C and adiponectin levels were shown to be significantly higher in CVE-free survivors. Multivariate analysis also demonstrated that female sex and high HDL-C plasma concentrations promoted CVE-free survival by an odds ratio that reached nearly 20-fold in risk reduction (Table 2). Previous epidemiological studies have already shown sex differences in CVD incidence, and described how occurrence of CVE, which usually appears 7 to 10 years later in women than men, could be partly due to the endogenous exposure to estrogens throughout the lifetime fertility interval. In fact, menopause transition is known to be associated with an increased risk of coronary heart disease [25]. However, our results demonstrate that female sex remains an important cardioprotective factor in patients with FH, even past 70 years (OR = 21.16 (2.48–180.4); *p* < 0.005), thus suggesting the presence of other than hormonal dependent pathways responsible for maintaining positive cardiovascular outcomes across this population. Other atherosclerotic risk factors could also influence the differences noted between men and women with respect to age, such as smoking, body fat distribution, systolic blood pressure and lipid profile. In the FH population, a difference of at least 20% of cumulative risk of a coronary event is observed by the age of 60 years between men and women who are not optimally treated. Moreover, if left untreated, men with FH could present symptoms of ASCVD in the fourth decade of their life, compared to those in women, which tend to appear 10 years later [26]. The biological markers identified here are also coherent with other previous findings describing HDL-C as an effective predictor of CVD risks [27]. It is well known that approximately 25% of patients with documented CVE have reduced levels of HDL-C (<35 mg/dL). In fact, decreasing LDL-C concentrations alone in patients with low levels of HDL-C has been described as not being sufficient to reduce cardiovascular risks [27]. Moreover, the correlation between female and HDL-C levels with regard to its association with CVE-free survival in FH was also described by Neil HA et al., revealing lower concentrations of HDL-C in HeFH subjects with CAD and this difference was notably more pronounced in women [28]. On the other hand, predictors of CVD in patients with FH were also evaluated across different studies, such as the Montreal cohort [29] or the SAFEHEART registry [30], which revealed that age, male sex, history of previous ASCVD, hypertension, smoking, low HDL-C levels, elevated LDL-C and Lp(a) were independent risk factors that modulate the incidence of CVD in patients with FH. Furthermore, male sex, untreated baseline LDL cholesterol > 6.47 mmol/L (250 mg/dL), hypertension, diabetes, statin therapy < 5 years, initiation of statin treatment over 30 years, positive genetic study for FH and obesity (BMI > 30 kg/m^2^) were also highlighted as risk factors associated with CVE in HeFH patients on statin therapy. These markers were prospectively analyzed from the Dyslipidemia Registry of the Spanish Society of Atherosclerosis [31].

Although adiponectin did not demonstrate a significant odds ratio of CVE-free survival (data now shown), plasmatic levels of this hormone were significantly higher in the CVE(-) (group 2 and 3) than the CVE(+) group (Table 1). It is well accepted that adiponectin acts as a key player in lipid metabolism and glucose homeostasis via insulin-sensitive tissues. More precisely, we and others have already shown that the circulating levels of adiponectin were associated with CAD [19], as well as several other aspects of the metabolic syndrome, including obesity, dyslipidemia and insulin resistance [32]. These effects are mostly due to the anti-inflammatory and anti-atherogenic properties of adiponectin that were demonstrated in several models, such as vascular smooth muscle cells [33], human aortic endothelial cells [34] and diabetic patients with/without CAD [35]. Altogether, these data highlight the importance of adiponectin to be considered as a marker of survival in the FH population. However, more efforts are still required in order to decipher the mechanism of action underlying the association of adiponectin to CVD risk reduction.

Among several biological factors that were identified in this study, one could expect that the nature of the FH-causing gene defect could also be classified as a contributory factor involved in the survival of FH subjects. Indeed, our previous findings showed significant differences in the relative odds ratio of being affected with CVD between carriers of receptor negative (>15 KB deletion) versus defective mutations (W66G) [36]. Moreover, comparisons between CVE (±) groups in the FH-confirmed genotype sample were also performed in this study and similar findings were obtained, with some determinants that did not reach statistical significance, clearly due to the limited number of subjects from the chosen cohort. However, temporality of association with CVD, often used in retrospective analyses, is the main limitation that the study encounters here. Such an issue should be taken into consideration mainly by following up on CVE reporting that occurs with CVE-free survivors, in order to validate the effect of those identified factors on the CVE incidence over time. Not to mention the sample size that remains relatively small in the FH survival cohort (group 3), which allow us to consider that some non-significant comparisons could have demonstrated important differences between groups if a larger sample was used for this cohort. Along with these limitations, one should also mention the number of patients with reported values for lipid parameters, which sometimes could reach less than 50% of the overall population (ex. Lp(a) levels), and this is mainly due to missing data from the patient’s medical file, therefore preventing comparisons from being significant between groups once statistical analyses were performed. In addition to FH-causing genes, research studies also showed that several other genetic variants could affect the phenotype of FH and allow subjects to be more or less prone to develop atherosclerosis. As for example, single point mutations of adiponectin (T45G) in exon 2 (rs2241766) and G276T in intron 2 (rs1501299) are genetic determinants shown to be closely associated with the CV risk path, most probably due to its effect on adiponectin plasma levels, particularly resulting in hypoadiponectinemia [37]. Based on the results obtained here that specifically show a significant association between elevated levels of adiponectin and CVE-free survival, we believe that further investigations are needed in order to confirm the correlation between these above-mentioned genetic variants of adiponectin and its observed plasma concentrations. Other potential candidate genes will also be studied, including Endothelin-1 (*EDN1*), Angiopoietin-like 3 (*ANGPTL3*) and Cholesteryl ester transfer protein (*CETP*). Most importantly, the exome sequencing of these FH subjects has been recently performed and ongoing analyses are currently being conducted with the goal of identifying novel genetic variants associated with CVE-free survival in FH.

### 4.2. Environmental Markers of Survival

Despite the lack of randomized controlled trials (RCTs) showing the effect of lipid-lowering treatment on reducing cardiovascular events in FH, pharmacologic therapies (e.g., statins, ezetimibe and anti-PCSK9 monoclonal antibodies) remain highly recommended for CVD risk management in this population. In fact, evidence showing the reduction in CVD-associated mortality and morbidity in patients using lipid-lowering medications was first described by the Scandinavian Simvastatin Survival Study (4S) trial with statins [38], and recently demonstrated by the CV outcome studies with the FOURIER (evolocumab) [39] and ODYSSEY (alirocumab) [40] trials. However, in addition to using lipid-lowering agents for treating hypercholesterolemia, guidelines also suggest for patients with FH to continuously follow a healthy lifestyle, from which long-term adherence could be beneficial to their cardiovascular health. Knowing that several lifestyle-related factors, such as smoking, low-quality diet with little or no physical activity contribute to an additional increase in atherosclerotic cardiovascular risk in FH, we thought of investigating the impact of environmental factors and lifestyle habits in determining the risk trajectory of CVE occurrence in FH. Indeed, we found that non-smoking was an important contributory factor associated with CVE-free survival in FH patients (Figure 2). Not to mention that non-smoking (OR = 10.18 (3.72–27.89); *p* < 0.0001) and high HDL-C (OR = 6.82 (2.04–22.84); *p* = 0.002) were also shown to be significant covariates associated with unexpected survival in FH. These findings correlate well with evidence found in the literature, where the CV risk association of smoking [41,42,43,44] and psychosocial stress [45,46] have been extensively studied.

## 5. Conclusions

**Figure 3:**
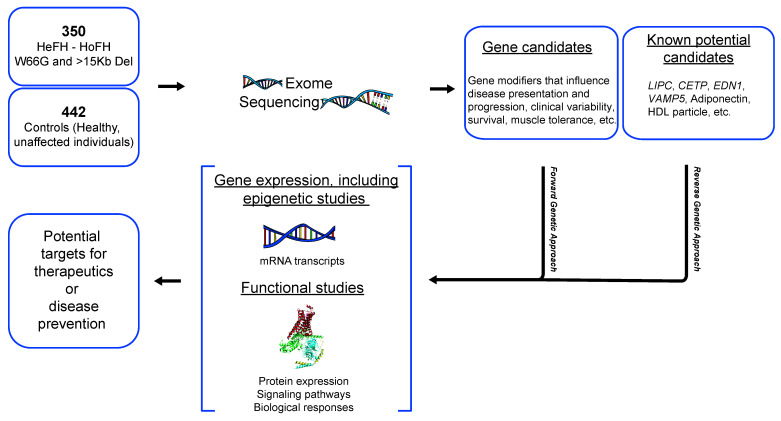
Ongoing exome sequencing analyses to identify genetic factors of CVE-free survival in FH. EDN1: Endothelin-1, CETP: Cholesteryl ester transfer protein, LIPC: Hepatic Lipase C, VAMP5: Vesicle associated membrane protein 5.

It is important to note that FH patients, especially those aged >70, who had never been hospitalized for a CVD-related event or for any revascularization procedure, have spent >40 years in the pre-statin era. Overall, less than 20% of FH French–Canadians were treated with a statin before the publication of the 4S study in 1994 [36,47], suggesting that the majority of FH subjects aged ≥70 from this study have spent at least five decades without conventional lipid-lowering therapies. Since then, they have all been treated with statins and results of the present study also confirm that the lipid-lowering regimen, although started later in life, contributes to CVE(-) FH survival. This study, which compares CVE(+) to CVE(-) FH subjects while considering elderly patients, is therefore unique in its kind and needs further investigation, in order to decipher the biological, environmental and genetic signature of these CVE-resistant factors. Despite the well-known association between FH and premature CVD, we were able to show that, beyond LDL-C levels, several factors (female, high HDL-C, elevated adiponectin and non-smoking) could contribute to CVE-free survival and probably constitute major targets for CVD risk prevention. In fact, adjusting for LDL-C levels in regression models showed similar results to the ones presented here. Such a parameter was not included in the analyses due to inconsistency in the collected LDL-C baseline, where patients could be either treated or not upon enrollment in the study. Among the identified markers, one should note that some of these were non-lipid parameters revealed as cardioprotective factors, which contributed to CVE-free survival in patients with FH. Interestingly, these findings highlight the need to consider non-lipid parameters, including lifestyle habits, for the CV risk management in FH. Systematic exploration of factors possibly involved in CVE-free survival in FH from this sample is ongoing. As mentioned above, a whole exome sequencing (using VCRome 2.1 design) was performed on 792 subjects with FH: a forward genetic approach will be used to identify gene modifiers that influence disease presentation and progression, survival or other clinical outcomes, and a reverse approach using burden testing and analyses in known potential candidates, such as hepatic lipase (*LIPC*), cholesteryl ester transfer protein (*CETP*), endothelin 1 (*EDN1*), vesicle associated membrane protein 5 (*VAMP5*), adiponectin and others will also be performed. Further investigations with regard to gene expression as well as functional analyses will also be planned (Figure 3). Identifying factors, including non-lipid parameters, that are susceptible to attenuate CVD development while facing elevated LDL-C exposure could lead to innovative therapies for FH patients with heart disease, whether dealing with non-optimal treatments or severe statin intolerance.

## Figures and Tables

**Table 1:** Identification of biological markers associated with cardiovascular events (CVE)-free survival in patients with familial hypercholesterolemia.

	CVE(+)	CVE(-)		*p*-value
	Men <55 years, Women <65 years (*n* = 458)	Men <55 years, Women <65 years (*n* = 1297)	≥70 years (*n* = 24)	
Female (%)	30.1	56.3 ^‡^	83.3 ^‡^	*p* < 0.0001
Age (years)	47.9 ± 9.2	33.7 ± 15.6 ^‡^	74.3 ± 3.6 ^‡^	*p* < 0.0001
Lipid-lowering treatment (%)	73.6	55.5 ^‡^	58.3	0.02
Type 2 Diabetes (%)	9.8	4.0 ^‡^	4.2	0.001
Hypertension (%)	35.8	14.9 ^‡^	50.0	*p* < 0.0001
Total Cholesterol (mmol/L)	8.47 ± 1.91	8.36 ± 1.61 ^a^	8.80 ± 1.56	NS
HDL-Cholesterol (mmol/L)	1.03 ± 0.31^b^	1.16 ± 0.33 ^c^^‡^	1.27 ± 0.49 ^#^	*p* < 0.0001
LDL-Cholesterol (mmol/L)	6.48 ± 1.86 ^d^	6.4 ± 1.50 ^e^	6.60 ± 1.36	NS
Non HDL-Cholesterol (mmol/L)	7.45 ± 1.96 ^f^	7.19 ± 1.61 *	7.53 ± 1.66	0.02
Total-C/HDL-C	9.21 ± 5.45 ^f^	7.80 ± 3.51 ^‡^	7.72 ± 2.90	*p* < 0.0001
VLDL-Cholesterol (mmol/L)	1.36 ± 1.29 ^g^	1.03 ± 1.04 ^h^ *	0.74 ± 0.53 ^i^	0.01
Triglycerides (mmol/L)	1.90 (1.30–2.70)^j^	1.50 (1.00–2.21) ^k^^‡^	2.20 (1.25–2.76)	*p* < 0.0001
VLDL-C/Triglycerides	0.40 (0.33–0.47)^l^	0.39 (0.33–0.46) ^m^	0.41 (0.28–0.49) *^n^*	NS
Apolipoprotein A1 (g/L)	1.23 ± 0.24 ^o^	1.35 ± 0.26 ^p^^‡^	1.30 ± 0.19^q^	*p* < 0.0001
Apolipoprotein B (g/L)	1.60 ± 0.39 ^r^	1.53 ± 0.29 ^s #^	1.53 ± 0.27 ^t^	0.01
Lp(a) (mg/dL)	24.40 (6.03–65.30) ^u^	19.20 (6.01–51.75) ^v^	-	NS
Adiponectin (mg/mL)	6.44 (4.30–9.06) ^w^	7.30 (4.96–10.14) ^x^ *	11.54 (9.50–16.43) ^y^^†^	*p* < 0.001

Data are mean ± SD, except for triglycerides, very low-density lipoprotein-cholesterol (VLDL-C)/triglycerides, Lp(a) and adiponectin that are shown as median (IQR). Analysis of data with skewed distribution was performed upon log_10_ transformation. Statistical significance across groups was analyzed by Pearson χ2 test for categorical variables and one-way ANOVA test for continuous variables. NS = *p*-value ≥0.05. Whenever significant, Bonferroni post-hoc analyses were performed, therefore comparing both age groups (men <55 years, women <65 years and ≥70) to premature CVE(+) (men <55 years and women <65 years). * *p*-value < 0.05, ^#^ *p*-value < 0.01, ^†^ *p*-value < 0.001, ^‡^ *p*-value < 0.0001. ^a^ *n* = 1292, ^b^ *n*= 457, ^c^ *n* =1292, ^d^ *n* = 442; ^e^ *n* = 1266, ^f^ *n* = 457, ^g^ *n* = 116, ^h^ *n* = 362, ^I^ *n* = 7, ^j^ *n* = 457, ^k^ *n* = 1294, ^l^ *n* = 127, ^m^ *n* = 402, ^n^ *n* = 7, ^o^ *n* = 223, ^p^ *n* = 472, ^q^ *n* = 8, ^r^ *n* = 219, ^s^ *n* = 628, ^t^ *n* = 9, ^u^ *n* = 215, ^v^ *n* = 397, ^w^ *n* = 268, ^x^ *n* = 695, ^y^ *n* = 12. CVE(+): with reported cardiovascular events; CVE(-): without reported cardiovascular events; HDL-C: high-density lipoprotein-cholesterol; LDL-C: low-density lipoprotein-cholesterol.

**Table 2:** Multivariate analysis of the contribution of biological variables to the relative odds of CVE-free survival in familial hypercholesterolemia (FH) patients.

	Men <55 years, Women <65 years			≥70 y		
	Model 1	Model 2 (Model 1 + HDL-C)	Model 3 (Model 2+ ApoA1)	Model 1	Model 2 (Model 1 + HDL-C)	Model 3 (Model 2+ ApoA1)
Sex (female)						
Odds ratio (95% CI)	3.31 (2.31–4.76)	2.84 (1.96–4.12)	2.73 (1.88–3.98)	21.16 (2.48–180.4)	11.45 (1.25–105.98)	11.97 (1.29–111.29)
*p*-value	<0.0001	<0.0001	<0.0001	0.005	0.031	0.029
HDL-C						
Odds ratio (95% CI)		2.91 (1.56–5.25)	1.79 (0.91–3.51)		9.78 (1.75–54.67)	18.08 (2.17–150.16)
*p*-value		0.001	0.091		0.009	0.007
Apolipoprotein A1						
Odds ratio (95% CI)			3.45 (1.48–8.01)			0.15 (0.01–3.65)
*p*-value			0.004			0.241

Reported cardiovascular events (CVE) were identified as the dependent variable and sex, high-density lipoprotein-cholesterol (HDL-C) and apolipoprotein A1 were included as independent variables for each corresponding model (*n* = 223 (group 1), *n* = 470 (group 2); *n* = 8 (group 3)). The type and dose (in quartiles) of lipid-lowering drugs were included in all models. Statistical significance is defined with a *p*-value <0.05.

**Table 3:** Multivariate analyses of the contribution of environmental variables to the odds of CVE-free survival in FH patients.

	Men <55 years, Women <65 years		≥70 years	
	Model 1	Model 2 (Model 1 + Daily Life Stress)	Model 1	Model 2 (Model 1 + Daily Life Stress)
Non-smoking				
Odds ratio (95% CI)	3.46 (2.53–4.74)	3.28 (2.39–4.51)	12.94 (3.54–47.26)	12.60 (3.40–46.68)
*p*-value	<0.0001	<0.0001	<0.001	<0.001
Daily life stress ^#^				
Odds ratio (95% CI)		2.14 (1.54–2.99)		2.73 (0.51–14.66)
*p*-value		<0.0001		NS

Reported cardiovascular events (CVE) were identified as the dependent variable and environmental factors (non-smoking and daily life stress) were included as independent variables for each corresponding model (*n* = 289 (group 1), *n* = 1008 (group 2); *n* = 15 (group 3)). Lipid-lowering drug treatment was included in all models. Statistical significance is defined with a *p*-value <0.05. # As compared to high-to-extreme stress.

## Data Availability

The data presented in this study are available on request from the corresponding author.
